# Guillain-Barré syndrome following bacterial meningitis: a case report and literature review

**DOI:** 10.1186/s12883-018-1211-4

**Published:** 2018-12-17

**Authors:** Li Ding, Zhongjun Chen, Yan Sun, Haiping Bao, Xiao Wu, Lele Zhong, Pei Zhang, Yongzhong Lin, Ying Liu

**Affiliations:** 1 Department of Neurology, the Second Hospital of Dalian Medical University, No. 467 Zhongshan Road, Shahekou District, Dalian City, 116027 Liaoning Province China; 2 Neuro-Interventional Ward, Dalian Municipal Central Hospital of Dalian Medical University, Dalian City, China; 3 Anesthesiology Department, Jilin University, China Japan Union Hospital, Changchun City, China; 4 Department of Nerve Electrophysiology, the Second Hospital of Dalian Medical University, Dalian City, China

**Keywords:** Guillain-Barré syndrome, Bacterial meningitis, Chronic suppurative otitis media

## Abstract

**Background:**

We reported a case of an adult that presented Guillain-Barré syndrome (GBS) after bacterial meningitis which was secondary to chronic suppurative otitis media (CSOM). To our knowledge, this is the first case involving an adult presenting with GBS following bacterial meningitis.

**Case presentation:**

A 46-year man with type 2 diabetes and otitis media (OM) suffered with fever, headache, and vomiting for 6 days. The patient’s neck stiffness was obvious and the Kernig and Brudzinski signs were produced. The result of cerebrospinal fluid (CSF) analysis and cytological examination of the CSF supported the diagnose of bacterial meningitis. On day 17 the patient felt numbness in both hands and feet, which gradually progressed to weakness of the limbs. Bladder dysfunction occurred, which required catheterization. The patient showed a tetraparesis with emphasis on the legs. The deep tendon reflexes of limbs were absent. The patient had peripheral hypalgesia and deep sensory dysfunction. The symptoms were possibly a result of GBS. Nerve conduction study showed that the F wave latency of the upper and lower limbs was prolonged, particularly the lower limbs. 8 days later the repeated nerve conduction study showed a low compound muscle action potential (3.3 mV) with a normal distal motor latency (14.2 ms) and a low motor nerve conduction velocity (34.3 m/s) in the tibial nerve. The patient still required assistance when walking 3 months after onset.

**Conclusions:**

GBS following bacterial meningitis is rare and limbs weakness in patients with bacterial meningitis was usually considered because of weakness. This case should serve as a reminder for clinical doctors that when a patient with bacterial meningitis complains about limbs numbness or weakness, GBS should be considered, especially when the patient had diabetes mellitus (DM) history.

## Background

Guillain-Barré syndrome (GBS) is an acute onset immune-mediated disorder of the peripheral nervous system. Patients with this disease usually manifest acute progressive weakness, hyporeflexia or areflexia, and elevated levels of protein in the CSF. Approximately two thirds of patients with GBS experienced a preceding infection or an antecedent event (ie. surgery or immunizations) a few weeks prior to neuropathy [1]. There are only two case reports where Guillain-Barre syndrome appeared following bacterial meningitis. One case is an 11-year-old boy who experienced Guillain-Barre syndrome following meningococcal meningitis [1] and the other case is a previously healthy 35 month old girl who had Listeria meningitis with selective spinal grey matter involvement and exhibited demyelination in the neurophysiological studies [2]. To our knowledge, this is the first case involving an adult presenting with GBS following bacterial meningitis.

## Case presentation

**Fig. 1:**
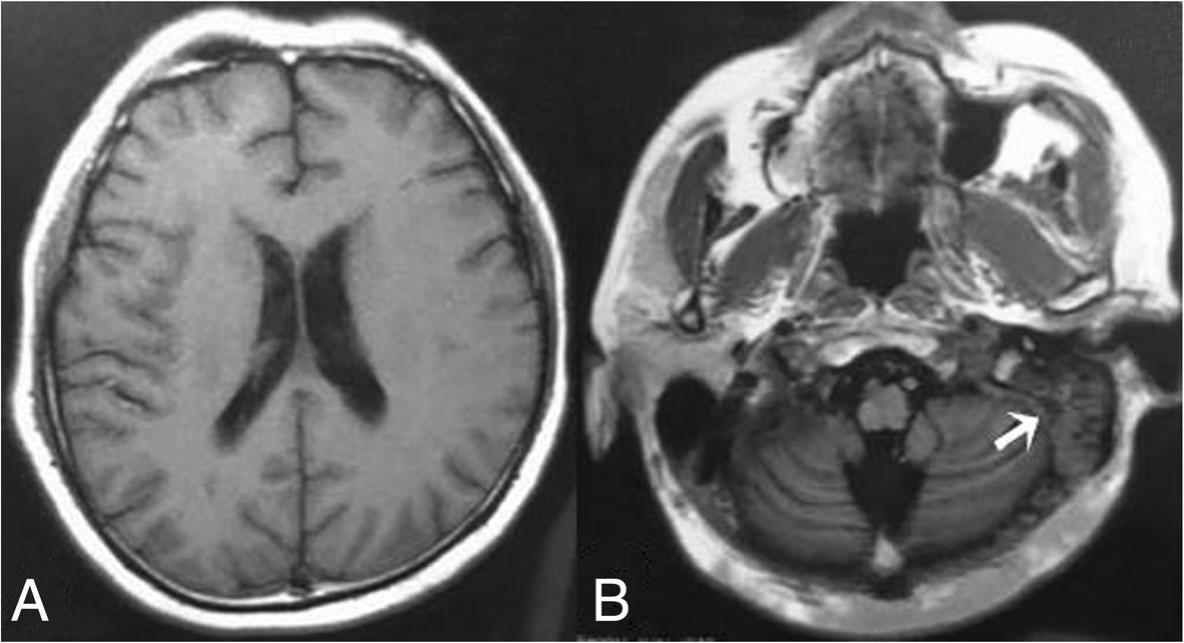
MRI at the junior hospital showed turbid sulcus (**a**) and OM in the left ear (arrows in **b**)

A 46-year man with type 2 diabetes (6 years) and OM (a few years) suffered with fever, headache, and vomiting for 6 days. There was purulent blood from the patient’s right ear. Magnetic resonance imaging (MRI) showed turbid sulcus (Fig. 1a) and OM of the left ear (Fig. 1. arrows in b). The lower class hospital treated with Cefperazone-Sulbactam for 3 days. The symptoms did not improve so the patient was transferred to the Second Hospital of Dalian Medical University (Dalian, China). The patient’s neck stiffness was obvious and the Kernig and Brudzinski signs were produced. The results of blood tests were showed in Table 1. In addition the glycosylated hemoglobin was 11.20%. The patient was diagnosed with diabetic ketoacidosis (DKA) and fluid replacement therapy was implemented. Lumbar puncture was operated on day 7 and the results of CSF analysis were showed in Table 2. The patient was diagnosed bacterial meningitis and treated with Ceftriaxone Sodium 3.0 g daily and vacocin vancomycin 1.0 g twice daily. Mannitol was used to dehydrate the cranial pressure. An otoscopy showed a bulging hyperemic tympanic membrane in the left ear and there were some bloody matter on the surface. The external auditory canal was swollen. The patient was diagnosed CSOM by an otolaryngologist and levofloxacin hydrochloride ear drops and erdosteine were prescribed. The lumbar puncture was repeated on day 14 and the results were showed in Table 2. Human albumin was added to dehydrate the cranial pressure. The blood culture was negative after cultured for 5 days. In order to reduce inflammatory reaction, dexamethasone 10 mg daily was added. On day 17 the patient felt numbness in both hands and feet, which gradually progressed to both legs and left arm. Bladder dysfunction occurred, which required catheterization. The patient showed a tetraparesis. Muscle strength in the right arm was grade 4 proximally and grade 4^−^ distally, in the left arm was grade 3 proximally and grade 2 distally, in the right leg was grade 2 proximally and grade 2^−^ distally, in the left leg was grade 0 both proximally and distally. The deep tendon reflexes of limbs were absent. The patient had distally pronounced hypalgesia and deep sensory dysfunction in arms and legs. Deep sensory dysfunction reached above wrists and was from the feet up to the knees. The symptoms were possibly a result of GBS. The blood culture was negative after an additional 5 days of culturing. IVIg was initiated at day 20. The patient’s symptoms improved, but his neck stiffness remained. The lumbar puncture was repeated on day 21 and the results were showed in Table 2. On day 22 the nerve conduction study showed that the F wave latency of the upper and lower limbs was prolonged, particularly the lower limbs (Table 3). On day 30 the repeated nerve conduction study showed a low compound muscle action potential (3.3 mV) with a normal distal motor latency (14.2 ms) and a low motor nerve conduction velocity (34.3 m/s) in the tibial nerve. The F wave latency of the lower limbs was prolonged (Table 4). Both nerve conduction studies supported the diagnosis of GBS. On day 33 acoustic impedance tests showed hearing loss in the left ear. On day 35 muscle strength in the right arm was grade 5 proximally and grade 5 distally, in the left arm was grade 4 proximally and grade 3 distally, in the right leg was grade 5 proximally and grade 5 distally, in the left leg was grade 4 proximally and grade 3 distally. Deep tendon reflexes were produced in the limbs. He had peripheral deep sensory dysfunction in the lower limbs. The patient was transferred to the local rehabilitation hospital. The patient still required assistance when walking 3 months after onset.

**Table 1:** The results of blood tests

Characteristics	Reference range	Day 6	Day 7	Day 8	Day 14	Day 16	Day 23	Day 28
WBC	× 10^9^/L	23.95	21.90	12.55	11.25	10.54	6.92	
NEUT%	40.00–75.00	90.3	89.30	82.50	75.30	90.30	72.80	
Fasting blood-glucose	3.9–6.1 mmol/L	16.39	refuse	17.1	14.3	7.0	11.8	8.7
Cl^−^	98.00–107.00 mmol/L	112.00	106.46	101.66	95.40	98.89	92.08	96.68
Urinary ketone body	negative	+++	++	negative	negative	negative		
pH	7.35–7.45	7.28	7.44	7.41		7.47		
PCO_2_	35-48 mmHg	15.90	27.20	25.00		34.60		
SBE	−1.5-3.0 mmol/L	−19.50	−5.10	−8.80		1.40		
cHCO_3_^−^	22.5–26.9 mmol/L	−11.80	20.50	18.30		25.70		

*Abbreviations*: *WBC* white blood cell, *NEUT* neutrophil, *PCO*_*2*_ partial pressure of carbon dioxide, *SBE* standard base excess, *cHCO*_*3*_^*−*^ actual bicarbonate

**Table 2:** The results of analysis of CSF samples obtained by lumbar puncture

Characteristics	Reference range	Day 7	Day 14	Day 21	Day 28
Appearance		hemorrhagic cloudy	hazy yellow	yellow transparent	yellow transparent
Opening pressure (lateral decubitus)	80–180 mmH_2_O	300	>330	265	250
WBC count	0–8 /μL	6400	320	92	11
L	%	5.00	19.00	46.00	
N	%	95.00	81.00	64.00	
Glucose	2.2–3.9 mmol/L	8.30	8.20	6.70	5.30
Total protein	120–600 mg/L	13,685.20	9857.70	5900.50	4545.50
Chlorides	120–132 mmol/L	119.15	107.80	112.39	117.42
CSF cultures	negative	negative	negative	negative	negative

*Abbreviations*: *WBC* white blood cell, *L* mononuclear cell, *N* multinuclear cell, *CSF* cerebrospinal fluid

**Table 3:** The results of nerve conduction study on day 22 after the onset

Nerve	Stimulation	Latency (ms)	Amp.	Velocity (m/s)	F-mean-latency	F-NO.%
Motor			(mV)			
Rt. peroneal	Ankle	3.5 (< 3.9)	6.2 (> 2.0)			
	Fibula (head)		5.1	40.2 (> 40.0)		
Lt. peroneal	Ankle	3.2 (< 3.9)	8.1 (> 2.0)			
	Fibula (head)		7.0	41.5 (> 40.0)		
Rt. tibial	Ankle	5.2 (< 5.1)	8.0 (> 4.0)		58.3 (< 53.9)	100.0 (> 70.0)
	Popliteal Fossa		8.0	43.8 (> 40.0)		
Lt. tibial	Ankle	4.3 (< 5.1)	5.1 (> 4.0)		59.9 (< 53.9)	100.0 (> 70.0)
	Popliteal Fossa		3.1	41.4 (> 40.0)		
Lt. median	Wrist	3.3 (< 4.0)	20.4 (> 7.0)		30.9(< 29.37)	100.0 (> 80.0)
	Elbow		20.1	51.0 (> 50.0)		
Lt. ulnar	Wrist	2.4 (< 3.1)	16.0 (> 7.0)		29.6 (< 29.1)	100.0 (> 80.0)
	Elbow		15.6	55.5 (> 50.0)		
Sensory			(μV)			
Rt. superficial peroneal	Lower leg	2.2	11.7 (> 5.0)	45.4 (> 40.0)		
Lt. median	Digit I	2.5	28.6(> 15.0)	52.0(> 43.4)		
	Digit III	3.0	21.9 (> 6.5)	46.6 (> 46.5)		
Lt. ulnar	Digit V	1.9	9.8 (> 7.0)	55.2(> 45.0)		
Lt. radial	Digit I	1.8	26.8(> 5.0)	52.7(> 40.0)		

*Abbreviations*: *Amp* amplitude, *Lt*  left, *Rt*  right, *F-NO.%* F wave occurrence rate

**Table 4:** The results of nerve conduction study on day 30 after the onset

Nerve	Stimulation	Latency (ms)	Amp.	Velocity (m/s)	F-mean-latency	F-NO.%
Motor			(mV)			
Lt. peroneal	Ankle	3.6 (< 3.9)	7.8 (> 2.0)		59.5 (< 54.1)	56.3 (> 70.0)
	Fibula (head)		7.0	40.9 (> 40.0)		
Lt. tibial	Ankle	4.0 (< 5.1)	4.6 (> 4.0)		57.6 (< 53.9)	100.0 (> 70.0)
	Popliteal Fossa		3.3	34.3 (> 40.0)		
Lt. median	Wrist	3.3 (< 4.0)	15.5 (> 7.0)		28.7(< 29.37)	95.0 (> 80.0)
	Elbow		14.6	51.0 (> 50.0)		
Lt. ulnar	Wrist	2.4 (< 3.1)	14.8 (> 7.0)		27.9 (< 29.1)	100.0 (> 80.0)
	Elbow		14.7	58.3 (> 50.0)		
Sensory			(μV)			
Lt. superficial peroneal	Lower leg	2.6	14.6 (> 5.0)	46.1 (> 40.0)		
Lt. sural	Ankle	2.7	29.0 (> 5.0)	45.1 (> 42.0)		
Lt. median	Digit I	2.5	32.9(> 15.0)	44.0(> 43.4)		
	Digit III	2.6	27.5 (> 6.5)	50.0 (> 46.5)		
Lt. ulnar	Digit V	1.9	20.6 (> 7.0)	52.6(> 45.0)		

*Abbreviations*: *Amp* amplitude, *Lt*  left, *Rt*  right, *F-NO.%* F wave occurrence rate

## Discussion and conclusions

GBS is a group of neuropathic diseases. Within 4 weeks of onset there are two-thirds of adult patients who have gastrointestinal tract or respiratory infection [3]. *Campylobacter jejuni* is found in 25–50% of the adult patients, and more frequently in Asian countries [4]. One study showed that *Mycoplasma pneumoniae* was found in 15% patients of GBS and was the second most common causative agent [5, 6]. Other causative agents included cytomegalovirus (CMV), Epstein-Barr virus [7], influenza A virus, *Listeria monocytogenes* [8], *Haemophilus influenza* [3], hepatitis E [9, 10], Zika and chikungunya [11]. A case report showed a case of Listeria meningitis in a previously healthy 35 month-old female with demyelination in the neurophysiological studies [2]. We reported a case of an adult that presented GBS after bacterial meningitis. Although the CSF was cultured four times and blood cultured twice, the results were all negative. The bacteria that caused this rare symptom remained unknown. The patient’s bacterial meningitis was secondary to CSOM. Causative agents of chronic CSOM mainly included *Proteus mirabilis*, *Pseudomonas aeruginosa*, *Staphylococcus aureus*, or anaerobic bacteria [12]. Bacterial meningitis is the most common intracranial complication of both acute and chronic OM. The common organisms caused this complication are H. influenzae Type B, *S. pneumoniae*, and Group A streptococcus [13]. It is likely that the organism that caused the symptoms in the patient were one of these.

Acute inflammatory demyelinating polyneuropathy and acute motor axonal neuropathy are the main phenotypes of GBS spectrum. The electrophysiological results of our patient revealed a demyelinating neuropathy with reduced motor nerve conduction velocity and prolonged F wave latency, which is in line with an acute inflammatory demyelinating polyneuropathy. The patient presented GBS symptoms on day 17 following bacterial meningitis. Critical illness polyneuropathy (CIP), which manifests as distally predominant limb weakness and reduced reflexes, should be distinguished. CIP is a sensory-motor axonal polyneuropathy that commonly develops in critically ill patients [14]. The electrophysiological characteristics of this neuropathy are reduced amplitude of the compound motor and the sensory nerve action potential, the near normal motor and sensory conduction velocities, the normal F-wave latency and denervation patterns on needle electromyograms [15]. The patient was critically ill but did not meet the criteria that were required for CIP. Thus, this patient appears unique in having GBS following bacterial meningitis.

An adult that presents with bacterial meningitis and GBS is not common. A history of diabetes mellitus (DM) could be critical, particularly since the patient’s blood glucose was not well controlled. Both DM type 1 and type 2 are associated with increased infection rates, particularly bacterial infections [16]. Infections in patients with diabetes are more likely to be severe [17]. This is likely the reason for the patient’s bacterial meningitis being so severe. A recent study which involved 85 patients that had acute polyradiculoneuropathy showed that DM type 2 was present in 32% patients. The three-month prognosis was worse in patients with both GBS and diabetes [18]. This could be explained in many ways. Some patients with DM may have pre-existing nerve injuries, and the onset of the GBS worsened the situation [18]. Diabetic patients have reduced rates of nerve regeneration, even before signs and symptoms of neuropathy appear. Once diabetic neuropathy appears, the capacity for nerve regeneration further decreases [19]. Diabetes could also increase the inflammation in GBS, since both diseases are associated with systemic inflammation [18].

In conclusion, GBS following bacterial meningitis is rare. However, our case shows that it should be considered in patients with bacterial meningitis suffering limb weakness and numbness, especially when the patient has DM history. Delayed diagnosis and treatment may result in poor neurological outcomes. This case should serve as a reminder for clinical doctors that when a patient with bacterial meningitis complains about limbs numbness or weakness, GBS should be considered, especially when the patient had DM history. IVIg should be started immediately after the diagnosis to prevent symptom progression.

## Abbreviations

CIP: Critical illness polyneuropathy
CMV: Cytomegalovirus
CSF: Cerebrospinal fluid
CSOM: Chronic suppurative otitis media
DKA: Diabetic ketoacidosis
DM: Diabetes mellitus
GBS: Guillain-Barré syndrome
LOS: Lipooligosaccharides
Lt: Left
MRI: Magnetic resonance imaging
OM: Otitis media
Rt: Right
